# Supplementary material for: Regulatory Crosstalk between Physiological Low O2 Concentration and Notch Pathway in Early Erythropoiesis
Source: Biomolecules. 2022 Apr 2;12(4):540. doi: 10.3390/biom12040540 (PMC9028139; doi:10.3390/biom12040540)
Supplement: Supplementary file 1 [file biomolecules-12-00540-s001.zip › biomolecules-1619916-supplementary.pdf]

Supplementary Material

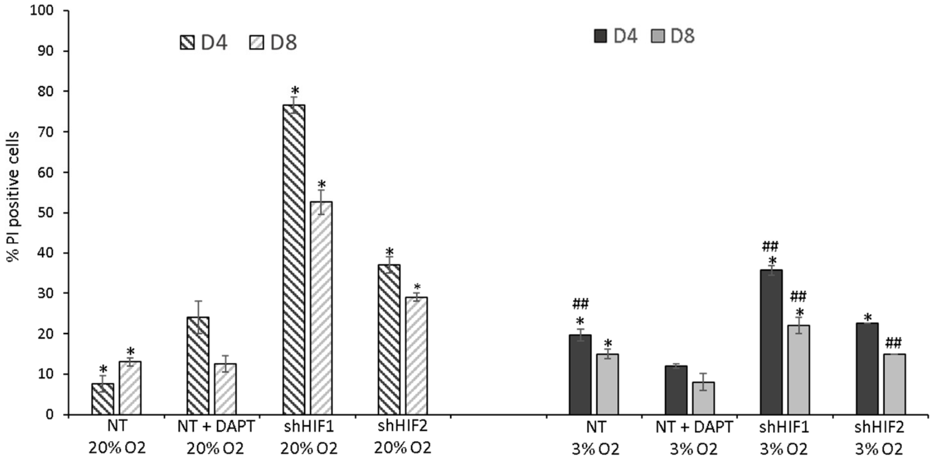

Supplementary Figure S1. Cell survival. Cell harvested at day 4 and day 8 culture propagated from non-transduced CD34+ cells in presence or absence of Notch γ-secretase inhibitor DAPT as well as transduced CD34+ cells with shHIF1α or with shHIF2α at 20 and 3% O2 were stained with propidium iodide (PI). The bars show percentage of PI positive cells representing dead cells. The values are given as a mean ± SD of three independent experiments. . Asterisks indicate a significant difference in the respect to NT condition, at $p < 0.05$ (*); Mann-Whitney test. NT, non-transduced CD34+ cells. p < 0.01 (##).

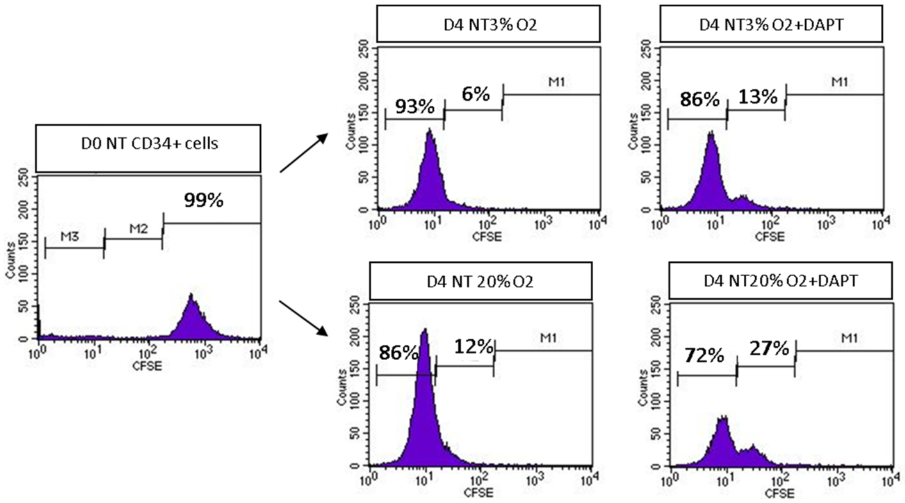

Supplementary Figure S2. Tracking cell division history by CFSE staining. Non-transduced CD34+cells were labeled with CFSE dye and culture for four days at 3%, and 20% O2 in the presence or absence the Notch signaling inhibitor DAPT. Histograms present CFSE intensity obtained by analyzing the cells at Day 0 (D0) and after 4 days (D4) of cultures. One representative out of three independent experiments is showed.

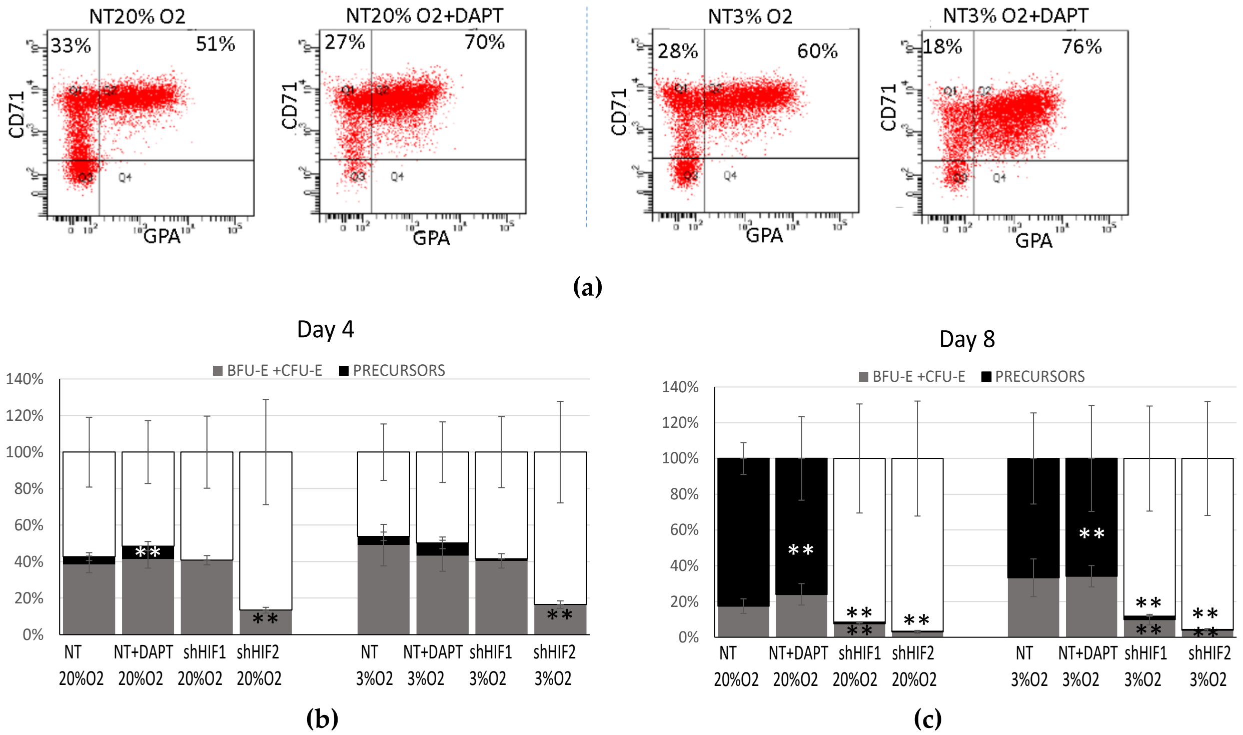

Supplementary Figure S3. Distribution of cellular types in time-course of erythroid culture. Eight-day erythroid cultures were propagated from non-transduced $CD34^+$ cells in presence or absence of Notch γ-secretase inhibitor DAPT as well as transduced $CD34^+$ cells with shHIF1α or with shHIF2α at 20 and 3% O2. At the figure **a)** scatter plot represent expression of transferrin receptor CD71 versus glycophorine A (GPA). One representative of five independent experiments is shown.The total number erythroid of progenitors was calculated by addition of the total number of BFU-E and CFU-E obtained at Day 4 **(b)** and Day 8 of culture **(c).** The total number of precursors cells was calculated based on the % of $CD71^+$/GPA^+ cells of total cells obtained at the same time-points. The number of non-erythroid cells (not exclusively committed to erythroid lineage) was calculated using a substraction : total number of cell – (BFU-E+ CFU-E + erythroid precursors). The bars represent the mean values ± SD of seven independent experiments. . Asterisks indicate a significant difference in the respect to NT condition, at $p < 0.05$ (*), $p < 0.01$ (**),Mann-Whitney test. NT, non-transduced $CD34^+$ cells.

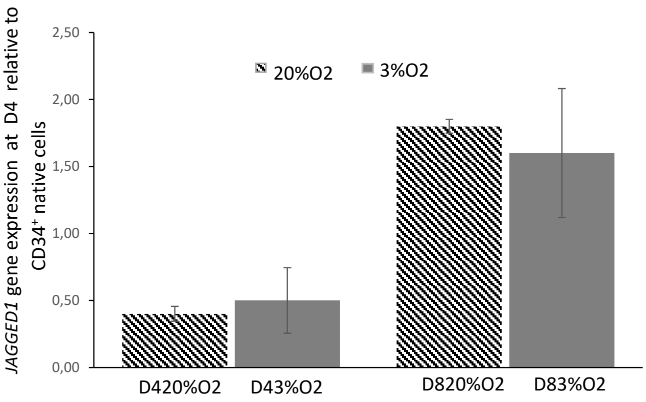

Supplementary Figure S4. Expression of Notch ligand *JAGGED1*. Gene expression was evaluated in erythroid cells obtained in day 4 and of culture of non–transduced $CD34^+$ cells at 20 and 3% O2 by real-time polymerase chain reaction analysis. Bars represent relative gene expression in respect to native $CD34^+$ CB cells. Data are showed as mean ± SD of three independent experiments

Gene	Primer	Sequence
hu β-aktin	forward	5'-ATTGGCAATGAGCGGTTC-3'
hu β-aktin	reverse	5'-GGATGCCACAGGACTCCAT-3'
hu HES-1	forward	5'-GATGTGAAGGCTTTCAAGACAGA-3'
hu HES-1	reverse	5'-GGAAAATGGCTTCCTTAGGC-3'
hu HEY-2	forward	5'-CCAGCAGTGCATCAGTATGTC-3'
hu HEY-2	reverse	5-'CAGGCACTTACGAAACACGA-3'
hu Jagged-1	forward	5'-TGCCAAGTGCCAGGAAGT-3'
hu Jagged-2	reverse	5'-GCCCCATCTGGTATCACACT-3’

Supplementary Table S1. Real-time polymerase chain reaction primers
